# Phylogenetic Diversity and Single-Cell Genome Analysis of “*Melainabacteria*”, a Non-Photosynthetic Cyanobacterial Group, in the Termite Gut

**DOI:** 10.1264/jsme2.ME17137

**Published:** 2018-03-29

**Authors:** Yuniar Devi Utami, Hirokazu Kuwahara, Takumi Murakami, Takahiro Morikawa, Kaito Sugaya, Kumiko Kihara, Masahiro Yuki, Nathan Lo, Pinsurang Deevong, Sasitorn Hasin, Warin Boonriam, Tetsushi Inoue, Akinori Yamada, Moriya Ohkuma, Yuichi Hongoh

**Affiliations:** 1 Department of Biological Sciences, Tokyo Institute of Technology Tokyo 152–8550 Japan; 2 Biomass Research Platform Team, RIKEN Biomass Engineering Program Cooperation Division, RIKEN Center for Sustainable Resource Science Tsukuba 305–0074 Japan; 3 School of Biological Sciences, University of Sydney Sydney, NSW 2006 Australia; 4 Department of Microbiology, Kasetsart University Bangkok 10900 Thailand; 5 College of Innovative Management, Valaya Alongkorn Rajabhat University under the Royal Patronage Pathum Thani 13180 Thailand; 6 School of Biology, Suranaree University of Technology Nakhon Ratchasima 30000 Thailand; 7 Graduate School of Fisheries and Environmental Sciences, Nagasaki University Nagasaki 852–8521 Japan; 8 Japan Collection of Microorganisms, RIKEN BioResource Center Tsukuba 305–0074 Japan

**Keywords:** symbiosis, insect, gut bacteria, single-cell genomics, cyanobacteria

## Abstract

Termite guts harbor diverse yet-uncultured bacteria, including a non-photosynthetic cyanobacterial group, the class “*Melainabacteria*”. We herein reported the phylogenetic diversity of “*Melainabacteria*” in the guts of diverse termites and conducted a single-cell genome analysis of a melainabacterium obtained from the gut of the termite *Termes propinquus*. We performed amplicon sequencing of 16S rRNA genes from the guts of 60 termite and eight cockroach species, and detected melainabacterial sequences in 48 out of the 68 insect species, albeit with low abundances (0.02–1.90%). Most of the melainabacterial sequences obtained were assigned to the order “*Gastranaerophilales*” and appeared to form clusters unique to termites and cockroaches. A single-cell genome of a melainabacterium, designated phylotype Tpq-Mel-01, was obtained using a fluorescence-activated cell sorter and whole genome amplification. The genome shared basic features with other melainabacterial genomes previously reconstructed from the metagenomes of human and koala feces. The bacterium had a small genome (~1.6 Mb) and possessed fermentative pathways possibly using sugars and chitobiose as carbon and energy sources, while the pathways for photosynthesis and carbon fixation were not found. The genome contained genes for flagellar components and chemotaxis; therefore, the bacterium is likely motile. A fluorescence *in situ* hybridization analysis showed that the cells of Tpq-Mel-01 and/or its close relatives are short rods with the dimensions of 1.1±0.2 μm by 0.5±0.1 μm; for these bacteria, we propose the novel species, “*Candidatus* Gastranaerophilus termiticola”. Our results provide fundamental information on “*Melainabacteria*” in the termite gut and expand our knowledge on this underrepresented, non-photosynthetic cyanobacterial group.

The termite gut is an environment that is populated by microbes, comprising protists, bacteria, archaea (6), and viruses (15, 31). Most of these microbes are unique to termites and extremely fastidious to cultivate (29). Extensive molecular ecological studies have been conducted to identify these uncultured microorganisms (13). Among them, 16S rRNA phylotype Rs-H34 (AB089123) was reported from the gut of the termite *Reticulitermes speratus*. This bacterium was phylogenetically related to *Gloeobacter violaceus*, a basal species in the phylum *Cyanobacteria* (12).

The phylum *Cyanobacteria* has been characterized primarily by the ability of its representatives to perform photosynthesis using H_2_O as the electron donor and generating oxygen (25). However, the discovery of cyanobacterial 16S rRNA genes from aphotic environments such as termite guts (12) and a bovine rumen (40) implied the presence of cyanobacteria with no photosynthetic capability. The presence of non-photosynthetic cyanobacteria was finally proven by Di Rienzi *et al.* (9); the authors reconstructed genomes phylogenetically related to cyanobacteria from the metagenomes of human fecal and groundwater samples. Soo *et al.* (2014) subsequently reconstructed several genomes closely related to those reported by Di Rienzi *et al.* (9) from the metagenomes of koala fecal samples and activated sludge and methanogenic sludge samples (36).

The candidate name “*Melainabacteria*” (“Greek nymph of dark water”) has been proposed for these bacteria, including phylotype Rs-H34 from the termite gut, in order to reflect their aphotic habitat (9). Since the 16S rRNA genes of these bacteria showed <85% sequence similarities with known phyla, “*Melainabacteria*” was initially considered to be a sister phylum of *Cyanobacteria* (9). However, Soo *et al.* (2014) demonstrated a robust phylogenetic relationship between *Cyanobacteria* and “*Melainabacteria*”; thus, they proposed “*Melainabacteria*” as a class within the phylum *Cyanobacteria*, sister to the class *Oxyphotobacteria* that comprises all photosynthetic cyanobacteria (36).

The latest study on this expanded *Cyanobacteria* phylum proposed three classes: *Oxyphotobacteria*, “*Melainabacteria*”, and “*Sericytochromatia*”, with the latter two lacking photosynthetic machinery (38). The only known cultivable species of “*Melainabacteria*” is *Vampirovibrio chlorellavorus*, which is a predator of *Chlorella vulgaris* and has been mistakenly classified to *Proteobacteria* (37). Four candidate orders have been proposed for the non-photosynthetic “*Melainabacteria*” class: “*Vampirovibrionales*”, “*Obscuribacterales*”, “*Gastranaerophilales*”, and “*Caenarcaniphilales*”, with the former two being microaerophilic and the latter two being strictly anaerobic groups (38).

Despite the discovery of melainabacterial 16S rRNA genes from termite guts (12, 44), previous studies did not focus on this bacterial group in the termite gut ecosystem. We herein investigated the distribution and phylogenetic diversity of “*Melainabacteria*” among various termite species and analyzed a single-cell genome of a melainabacterium obtained from the gut of the interface-feeding higher termite *Termes propinquus* (family Termitidae; subfamily Termitinae). Our results provide fundamental information on melainabacteria in the termite gut and expand our knowledge of this still underrepresented bacteria group.

## Materials and Methods

### Sample collection and DNA extraction

Seventy-seven samples consisting of 60 termite and eight cockroach species were collected from six different districts (Table S1). The entire guts of 5 to 20 workers were removed with sterilized forceps for each termite sample and placed in a 1.5-mL tube on ice. DNA was extracted using a combination of the Isoplant II Kit (Nippon Gene, Tokyo, Japan) and DNeasy Tissue or Plant Kit (Qiagen, Hilden, Germany) as described previously (42). Regarding cockroach specimens, the entire gut was removed from one adult insect. Due to the large gut size, it was divided into three parts (foregut, midgut, and hindgut), except for *Blattella germanica*. DNA extraction from each part was performed in the same manner as in termites, and DNA samples were then mixed.

In addition to morphological characteristics, the mitochondrial cytochrome oxidase II (COII) gene was used to identify the host insect species. PCR amplification with the primers COII-FW-AtLeu and COII-RV-B-tLys (Table S2) and Sanger sequencing of the products were performed as described previously (16).

### Amplicon sequencing of 16S rRNA genes

The concentration of purified DNA samples was adjusted to 2 ng μL^−1^ with TE buffer (10 mM Tris-HCl pH 8.0, 1 mM EDTA), and samples were used as templates for PCR at a final concentration of 0.2 ng μL^−1^. 16S rRNA genes were amplified by PCR with the *Bacteria*-specific 341F and 806R primer set (Table S2), which targets the V3 and V4 regions (approx. 400 bp). PCR conditions and the purification of amplicons were as described previously (16). Paired-end sequencing was conducted on the Illumina MiSeq platform with the MiSeq Reagent Kit V3 as described previously (26). Data from a *Stolotermes victoriensis* sample were obtained in our previous study (16).

The generated sequence reads were trimmed, quality-filtered, and sorted into operational taxonomic units (OTUs) using the DADA2 v1.2.1 program package (7). Trimming and quality filtering were performed using the fastqPairedFilter function with the following parameters: truncLen=280 bp and 230 bp for forward and reverse sequence reads, respectively; trimLeft=5 bp for forward sequence reads; maxN=0; truncQ=2; and maxEE=2 and 5 for forward and reverse sequence reads, respectively. Subsequent dereplication, sequence error correction, and merging read pairs were performed with the default settings. After the elimination of merged sequences <350 bp, chimeric sequences were identified and removed using default settings. Following these procedures, the resulting OTUs represented unique sequences and were not defined with sequence similarity (7); thus, the developers of the DADA2 program have proposed the term “exact sequence variants (ESVs)” instead of “OTUs” (8); however, we used the latter in the present study.

### Phylogenetic analyses based on 16S rRNA gene and COII amino acid sequences

The OTUs generated by DADA2 were phylogenetically classified using SINA v1.2.11 (33) with the database SILVA SSURef NR99 release 128 with default settings, except for minimum similarity ≥80%. OTUs assigned to *Eukarya*, *Archaea*, mitochondria, chloroplasts, and *Blattabacterium*, a fat-body endosymbiont of cockroaches and *Mastotermes darwiniensis*, were removed. We also removed OTUs that did not align with other 16S rRNA sequences. The retained OTUs and reference sequences retrieved from the SILVA database were aligned using ARB software (21) with manual corrections. A neighbor-joining tree was constructed using MEGA7 (18).

A phylogenetic tree of the host insects was constructed based on the deduced amino acid sequences of the COII genes, using MEGA-CC (17). Sequences were aligned using MUSCLE (11) with ambiguously aligned sites being removed by Gblocks (41).

### Single cell sorting

The guts of two workers of *T. propinquus*, which were collected at Sakaerat Environmental Research Station, Nakhon Ratchasima Province, Thailand, were homogenized with a mortar and suspended in 800 μL of sterile solution U buffer (43). The mixture was filtered using a CellTrics Filter with 5-μm pores (Sysmex Partec, Görlitz, Germany), and then centrifuged at 9,100×*g* at 4°C for 5 min. The pellet was collected and resuspended in 400 μL of solution U. The centrifugation and resuspension steps in solution U, kept for 3 min, were repeated three times. The resulting pellet was treated with RQ1 RNase-Free DNase (Promega, Madison, WI, USA) (320 μL of sterile double-distilled water [DDW], 40 μL of 10×RQ1 buffer, and 40 μL DNase I) at 30°C for 30 min to digest extracellular DNA. The reaction mixture was centrifuged at 7,300×*g* at 4°C for 3 min. Additional washing steps with centrifugation in solution U at 9,100×*g* at 4°C for 3 min were repeated three times. The pellet was resuspended in 490 μL of solution U.

The resulting *ca.* 500-μL suspension, combined with 5 μL of fluorescent dye FM1-43 (Molecular Probes, Eugene, OR, USA), was subjected to fluorescence-activated single-cell sorting on BD FACSJazz™ (BD Biosciences, San Jose, CA, USA). Conditions for cell sorting (*e.g.*, flow speed, gating based on scattered light, and fluorescence intensity) were optimized by checking the presence of bacterial cells under epifluorescence microscopy. Single cells were sorted to 1 μL of ultraviolet (UV)-treated DDW placed in a UV-treated 96-well PCR plate (AB-0900; Thermo Fisher Scientific, Waltham, MA, USA) and then stored at −80°C.

### Whole genome amplification (WGA) and taxonomic identification of products

WGA was performed according to Sato *et al.* (34) using the Illustra GenomiPhi V2 Kit (GE Healthcare, Chicago, IL, USA). A stored 96-well plate with sorted single cells was thawed and briefly centrifuged. One microliter of lysis buffer (0.4 M KOH, 10 mM EDTA, 100 mM dithiothreitol) was added to each well, followed by brief centrifugation and an incubation on ice for 10 min. One microliter of neutralization buffer (0.4 M HCl, 0.6 M Tris-HCl, pH 7.5) was then added. Samples were centrifuged and mixed with 3.5 μL of Sample Buffer in the kit. A reaction mixture (0.5 μL enzyme solution and 4.5 μL Reaction Buffer in the kit) was added to the samples, which were then incubated at 30°C for 20 h in a PCR thermal cycler. All these reagents were UV-treated before use (46). Thereafter, an incubation at 65°C for 10 min was performed to inactivate the enzyme.

An aliquot of each WGA product was diluted 100-fold with TE buffer and used as a template for PCR amplification of the 16S rRNA gene. PCR was performed using *TaKaRa EX Taq* DNA polymerase (Takara Bio, Otsu, Japan) with the *Bacteria*-specific 27F-mix and 1390R primer set (Table S2). The PCR program was as follows: initial denaturation at 95°C for 1 min, 25 cycles of denaturation at 95°C for 15 s, annealing at 50°C for 30 s, elongation at 72°C for 1 min, and a final extension at 72°C for 5 min. Sanger sequencing was performed as described previously (16). The degree of contamination was judged from the quality of the 16S rRNA sequence as an initial screening, as described previously (49).

### Single-cell genome sequencing, assembly, and purity check

Among the surveyed single-cell genomes, a WGA product from a “*Melainabacteria*” cell was found and designated phylotype Tpq-Mel-01. A sequencing library for this sample was prepared using the TruSeq DNA Sample Preparation Kit according to its protocol for low DNA input (Illumina, San Diego, CA, USA). Sequencing was performed on the Illumina MiSeq platform with its Reagent Kit V3. Sequence reads were processed for adapter and quality trimming using the cutadapt (22) and PrinSeq programs (35). Qualified reads were assembled using SPAdes 3.5.0 to construct contigs (5).

Gene finding was conducted using the MiGAP (http://www.migap.org/) and RAST server (4). Contigs that contained one or more protein-coding sequences (CDS) showing the highest amino acid sequence similarity to those of known melainabacterial genomes (9, 36) in BLASTp searches against the NCBI non-redundant (nr) protein sequence database were retained for further analyses as “trusted” contigs. In addition, a tetranucleotide frequency-based emergent self-organizing map (ESOM) analysis was conducted as described previously (10) using the tetramerFreqs program (https://github.com/tetramerFreqs) with default settings, except for the min. length=500 bp and window size=5,000 bp. Since putatively contaminating contigs were closely related to either *Eubacterium siraeum* (NC_021011) or *Paenibacillus mucilaginosus* (NC_015690), based on a BLASTn search result against the NCBI nr nucleotide database, these genomes were selected as reference sequences. Only contigs that clustered with the “trusted” contigs in the ESOM analysis were retained.

### Characterization of genomes and phylogenomics

The functional annotation of genes was conducted using the MiGAP and RAST server, and metabolic pathways were reconstructed with the KEGG automatic annotation server (KAAS) (24). The results obtained were manually checked by BLASTp searches against the NCBI nr protein sequence database. Genome size and completeness were estimated based on the presence of 83 single copy genes conserved in the known “*Melainabacteria*” genomes (36). These genes were identified using HMMER 3.1b2 software (http://hmmer.org/). The overall nucleotide sequence similarity between the Tpq-Mel-01 genome and other known melainabacterial genomes was calculated using the Genome-to-Genome Distance Calculator with formula 2 for draft genomes (3). The identification of genes coding for secretion systems or type IV pili was achieved using TXSScan (1).

A maximum likelihood tree was constructed using FastTree v2.1.9 (32) based on the concatenated amino acid sequences of a subset of the above 83 single copy gene markers, detected using AMPHORA2 software (48). Concatenated sequences were then aligned using MUSCLE and edited using Gblocks.

### Fluorescence in situ hybridization (FISH)

A probe, MelTpq-646 (Table S2), specific to the 16S rRNA of phylotype Tpq-Mel-01, was designed using ARB and labeled with Texas Red at the 5′ end. The probe has at least three mismatches against sequences in the SILVA database. The entire guts were removed from two *T. propinquus* workers, and gut contents were suspended in solution U and incubated at room temperature for 30 min in order to precipitate and remove large particles. The supernatant was collected into a new tube and fixed with 4% paraformaldehyde at 4°C for 5 h. The mixture was centrifuged at 12,000×*g* for 10 min, and the pellet was washed three times with sterile DDW during centrifugation. The pellet was resuspended in 100 μL DDW, spotted onto MAS-GP slide glasses (Matsunami, Osaka, Japan), and air-dried. Subsequent procedures were as described previously (27) with hybridization at 55°C for 1 h. Specimens were enclosed with the *SlowFade*™ Gold Antifade Mountant with 4,6-diamidino-2-phenylindole (DAPI) (Thermo Fisher Scientific). Observations were conducted under an Olympus BX51 epifluorescence microscope (Olympus, Tokyo, Japan). Cell sizes were measured with ImageJ software (https://imagej.nih.gov/ij/).

### Database accession numbers

The Tpq-Mel-01 genome assembly has been deposited at the DDBJ under accession numbers BEIT01000001–165. Representative sequences of the melainabacterial OTUs of the 16S rRNA gene will appear under accession numbers LC322338–421.

## Results and Discussion

### Abundance and phylogenetic diversity of “*Melainabacteria*” in termite guts

**Fig. 1:**
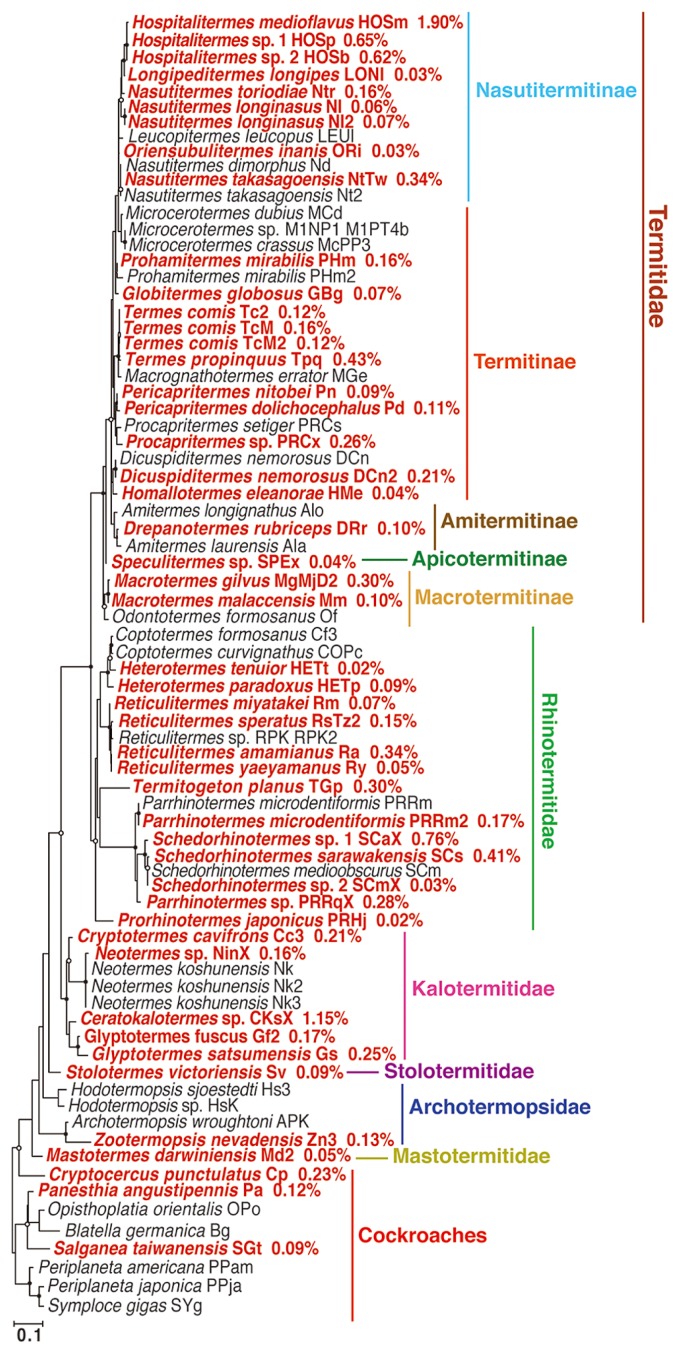
Maximum likelihood tree of termites and cockroaches used in the present study based on deduced amino acid sequences of the mitochondrial cytochrome oxidase II gene. The 16S rRNA genes of “*Melainabacteria*” were detected from the insect species shown in bold and red. The frequency of melainabacterial sequences is indicated for each sample. A total of 200 amino acid sites were used with the mtRev+G+I substitution model and 500 bootstrap resamplings.

The 16S rRNA genes of “*Melainabacteria*” were detected in 48 out of the 77 analyzed termite and cockroach gut samples (Fig. 1 and Table S1). These included 45 out of 60 termite and three out of eight cockroach species. A total of 81 melainabacterial OTUs were identified; 79 were affiliated with a strictly anaerobic group, order “*Gastranaerophilales*”, and two were classified to a microaerophilic group, order “*Obscuribacterales*” (Fig. S1, S2, and S3). The relative abundance of these melainabacterial OTUs was consistently low, ranging between 0.02% and 1.90% (Fig. 1). Melainabacterial sequences were found from all of the six termite families, including five higher termite subfamilies, examined in the present study (Fig. 1 and Table S1).

Most of the melainabacterial OTUs from the termites and cockroaches appeared to cluster together and have never been detected from other environments (Fig. S1 and S2); these melainabacteria may specifically inhabit termite and cockroach guts. One to five OTUs were identified from each termite or cockroach species (Table S1). Multiple OTUs from a single host species did not necessarily form a monophyletic cluster (Fig. S2); one host species accommodates distantly related melainabacteria.

Each of sixty-nine out of the 81 melainabacterial OTUs was identified only from a single termite or cockroach species, implying that most melainabacterial OTUs were unique to respective termite or cockroach species. As exceptions, the remaining 12 OTUs were detected in two different termite or cockroach species (Fig. S2). Among them, OTU-00856 and 02378 were identified from two of the three *Hospitalitermes* species, and OTU-06201 was from two *Reticulitermes* species. Closely related termite species were likely to possess closely related melainabacterial OTUs, although the short sequence reads did not resolve the relationships with reliable bootstrap confidence values (Fig. S2). Other than the aforementioned, the other nine OTUs that were identified from two phylogenetically distinct termite or cockroach species (Fig. S2) may have been derived from the artificial “cross-talk” of the index reads on the Illumina sequencing platform (47) or the contamination of DNA during experiments. In these cases, the number of termite and cockroach species with melainabacteria detected may decrease from 48 to 44.

### Genomic features of phylotype Tpq-Mel-01 and its phylogenetic relationships to known melainabacteria

**Fig. 2:**
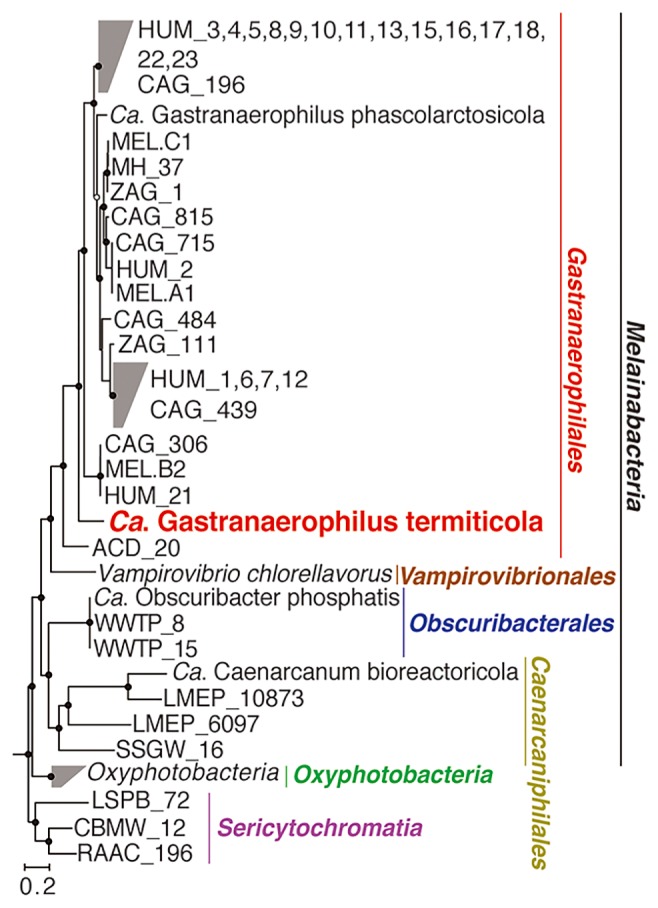
Phylogenetic position of phylotype Tpq-Mel-01 based on concatenated amino acid sequences of single copy genes conserved among the dataset. A maximum likelihood tree was constructed with the LG+G substitution model and 100 bootstrap resamplings. A total of 1,910 amino acid sites were used. Bootstrap confidence values ≥50% are shown by open circles and ≥70% by closed circles. *Chloroflexus aurantiacus* (GCF_000018865), *Roseiflexus castenholzii* (GCF_000017805), and *Anaerolinea thermophila* (GCF_000017805) of the phylum *Chloroflexi* were used as the outgroup and omitted from the tree.

The total sequence length of the single-cell genome of phylotype Tpq-Mel-01 was 960,865 bp, packed in 165 contigs. The GC content was 42.5%, and the largest contig size was 45,799 bp. The estimated genome completeness and predicted genome size were 61% and 1.6 Mbp, respectively. The conserved single copy marker genes used for the estimation had no sequence variations; there were no signs of contaminating sequences in the final contig set. This draft Tpq-Mel-01 genome encoded 1,079 putative CDSs, a single rRNA operon, and 22 tRNAs. The genome had a higher GC content and smaller predicted size than the genomes of other “*Gastranaerophilales*” members (Table 1). The average nucleotide sequence similarity of alignable genome regions between Tpq-Mel-01 and other genome-reconstructed melainabacteria was lower than 93% (Table S3). A phylogenomic tree showed that Tpq-Mel-01 was a sister to the monophyletic group comprising sequences from human and koala fecal samples in the order “*Gastranaerophilales*” (Fig. 2). Although the 16S rRNA gene sequence of phylotype Tpq-Mel-01 was not recovered by amplicon sequencing from the *T. propinquus* gut sample, possibly due to its very low abundance, it formed a monophyletic cluster with sequences from several higher-termite species, including *T. propinquus* (Fig. S2).

### Predicted functions of phylotype Tpq-Mel-01

**Fig. 3:**
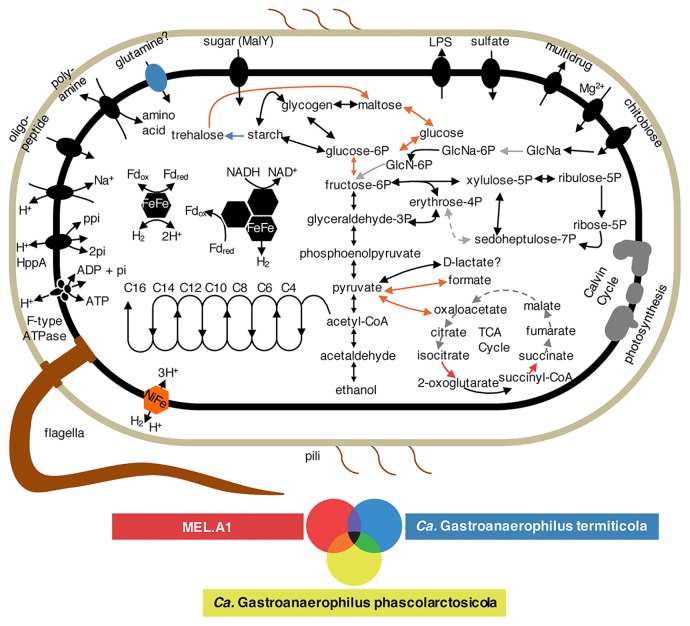
Predicted metabolic pathways of phylotype Tpq-Mel-01 in comparison with “*Ca.* Gastranaerophilus phascolarctosicola” and MEL.A1. Pathways found in more than one genome are highlighted with colors indicated below the pathway map. Missing pathways are shown in gray.

The Tpq-Mel-01 genome shared many features with other members of “*Gastranaerophilales*”. As representatives of the known “*Gastranaerophilales*” members (Fig. 2), the genomes of “*Candidatus* Gastranaerophilus phascolarctosicola” from koala feces (36) and the melainabacterium, MEL.A1, from human feces (9) were selected, based on their high genome completeness, for comparisons with the Tpq-Mel-01 genome. The gene sets required for photosynthesis, CO_2_ fixation, and respiratory chains were not found in the Tpq-Mel-01 draft genome, as observed in the other two genomes (Fig. 3). Similarly, only partial gene components of the tricarboxylic acid (TCA) cycle were found. However, the three genomes retained the *rpaA* and *rpaB* genes that regulate circadian rhythms in photosynthetic cyanobacteria, as reported previously (9). The genomes shared genes involved in the biosynthesis of the Gram-negative-type cell wall and S-layer-like components (9, 36).

The Tpq-Mel-01 genome encoded most genes involved in the pathways for glycolysis, gluconeogenesis, and pentose phosphate biosynthesis, similar to other “*Gastranaerophilus*” genomes (Fig. 3) (9, 36). The Tpq-Mel-01 bacterium and the other two “*Gastranaerophilus*” bacteria possessed a gene coding for a sugar transporter (MalY) of the major facilitator superfamily and genes for a putative chitobiose transporter (ChiFG). The genome also encoded β-N-acetylhexosaminidase (NagZ) to hydrolyze chitobiose into N-acetylglucosamine and N-acetylglucosamine-6-phosphate deacetylase (NagA), which participates in the conversion of N-acetylglucosamine to fructose-6-phosphate. Thus, Tpq-Mel-01 may use sugars (substrate unidentified) and chitobiose as the main carbon and energy sources and ferment them to ethanol and, possibly, D-lactate; it may produce ATP only by substrate-level phosphorylation. Thus, F_o_F_1_-type ATPase may be used to create a proton gradient by hydrolyzing ATP to ADP (Fig. 3). Tpq-Mel-01 has potential to store glycogen, similar to the other “*Gastranaerophilus*” members (Fig. 3). Only partial pathways were found for the biosynthesis of amino acids and cofactors, possibly due to the incomplete genome assembly (Fig. S4). The genes required for nitrogen fixation were not found in the Tpq-Mel-01 genome or those of any other “*Gastranaerophilus*” members, except for the melainabacterium, ACD_20, obtained from groundwater (9). Nitrogen may be taken up by importing oligopeptides and amino acids (Fig. 3).

Previous studies suggested that members of “*Gastranaerophilales*” possess (NiFe) and (FeFe) hydrogenases (9, 36). Genes coding for a trimeric (FeFe) hydrogenase (HydABC), a monomeric (FeFe) hydrogenase, and maturase units (HydE and HydG) were found in the Tpq-Mel-01 genome. Although the *hydG* gene was split into two parts by a frameshift and the *hydA* gene was split into three by a frameshift and inversion, difficulties are associated with discriminating a pseudogene from an artifact caused during WGA in single-cell genomics (20).

Previously reported genomes of “*Gastranaerophilales*” contained partial gene sets for the construction of flagella and pili (9, 36), whereas photosynthetic cyanobacteria lack flagella and utilize secreted polysaccharides and type IV pili for motility (45). Di Rienzi *et al.* (2013) suggested that melainabacteria from human fecal samples have the ability to move with flagella (9), whereas Soo *et al.* (2014) inferred that those from koala feces are non-motile due to the incompleteness of the flagella gene set (36). In the case of Tpq-Mel-01, its genome encoded more genes for flagellar assembly and chemotaxis than previously reported melainabacteria (Fig. S5); thus, it is highly possible that the bacterium moves using flagella. In addition, the Tpq-Mel-01 genome contained genes related to type IV pili twitching motility: *pilA* for pilin, *pilB*, *pilC*, and *pilT* for the inner membrane motor subcomplex, and *pilD* for processing prepilin (Fig. S6) (14).

### FISH analysis of phylotype Tpq-Mel-01

**Fig. 4:**
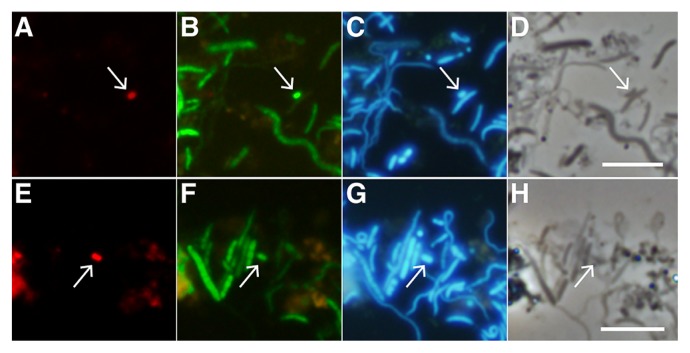
*In situ* detection of phylotype Tpq-Mel-01 in the gut content of *Termes propinquus*. Two examples (A–D and E–H, respectively) are shown. (A, E) FISH images of Tpq-Mel-01 cells detected with the probe MelTpq-644 labeled with Texas Red (red). (B, F) FISH images of bacterial cells detected with the probe EUB338 labeled with 6-carboxyflorescein (green). (C, G) DAPI images (blue). (D, H) Phase-contrast images. Arrows indicate Tpq-Mel-01 cells. Bars=5 μm. See the Results and Discussion sections for a discussion on the specificity of the probe MelTpq-644.

FISH analyses using the probe MelTpq-646 rarely, but successfully detected cells in the gut content of *T. propinquus*; two examples are shown in Fig. 4. We checked the specificity of the probe against melainabacterial OTUs obtained by amplicon sequencing in the present study, and found that it also completely matched the sequence of OTU-12005-PRCx from the termite *Procapritermes* sp. in addition to phylotype Tpq-Mel-01. Furthermore, the probe contained only one mismatch with the corresponding 16S rRNA gene region of three OTUs that constituted a monophyletic cluster with phylotype Tpq-Mel-01: OTU-07911-HOSp from *Hospitalitermes* sp., OTU-14642-Pn from *Pericapritermes nitobei*, and OTU-19367-Tpq from *T. propinquus* (Fig. S2). Sequence similarities among Tpq-Mel-01 and these three OTU sequences ranged between 96.7 and 99.4%, and the probe hybridized with all of them under the FISH conditions used in the present study according to *in silico* predictions with the DINAMelt web server (http://unafold.rna.albany.edu/?q=DINAMelt). Thus, it is possible that the detected cells were either of Tpq-Mel-01 or OTU-19367-Tpq, which shared 96.7% sequence identity.

The morphological characteristics of the detected cells were described below. This is, to the best of our knowledge, the first detection of cells of “*Gastranaerophilales*” members by a FISH analysis. Based on genome sequence and FISH data, we propose the novel species, “*Candidatus* Gastranaerophilus termiticola”, for phylotype Tpq-Mel-01 and its close relatives.

### Description of “*Candidatus* Gastranaerophilus termiticola”

*Gastranaerophilus termiticola* (ter.mi.ti’co.la, L. gen. n. *termitis*, termite; L. suffix–*cola* inhabitant, dweller); *G. termiticola*, a bacterium from genus *Gastranaerophilus* living in termite guts. Cells are rod shaped with dimensions of 1.0±0.2 μm by 0.5±0.1 μm (*n*=7). Potentially motile with flagella and type IV pili. The bacterium may ferment sugars and chitobiose to H_2_, ethanol, and D-lactate. It specifically inhabits the termite gut. This assignment is based on the 16S rRNA gene sequence, which was found in a contig (BEIT01000002), and specific hybridization with the oligonucleotide probe MelTpq-646 (Table S2).

## Conclusion

Melainabacteria were rare, but commonly present in the guts of diverse termite species. The relationship with the termite host is mostly specific; this minor group may be vertically transmitted via proctodeal trophallaxis as suggested in protists and dominant bacterial groups in the termite gut (2, 13, 23, 28, 39). In our single-cell genome analysis of “*Ca.* Gastranaerophilus termiticola” phylotype Tpq-Mel-01, no evidence was found to support the bacterium being directly involved in the hydrolysis of cellulose or hemicellulose, nitrogen fixation, the recycling of uric acids, reductive acetogenesis, or hydrogen oxidation, which are considered to be critical roles of gut bacteria for mutualism with the termite host (6, 13, 19, 30). Thus, melainabacteria in the termite gut appear to be commensal, but still comprise a part of the microbiota specific to the guts of termites and cockroaches.

## Supplementary Material



## Figures and Tables

**Table 1:** Genome features of phylotype Tpq-Mel-01 and other melainabacteria.

Proposed taxonomic name	GC (%)	CDS	Completeness^a^	Genome size (Mbp)^b^	Origin	Ref.
**Tpq-Mel-01 (“*Ca.* Gastranaerophilus termiticola”)**	**42.5**	**1,079**	**61%**	**0.96 (~1.6)**	**termite gut**	**this study**
“*Ca.* G. phascolarctosicola”	38.5	1,838	100%	1.8	koala gut	36
MEL.A1	32.9	1,879	100%	1.9	human gut	9
ACD20	33.5	2,455	100%	2.7	aquifer	9
“*Ca.* Caenarcanum bioreactoricola”	27.5	1,917	100%	1.8	bioreactor	36
“*Ca.* Obscuribacter phosphatis”	49.4	4,392	99%	5.5 (~5.6)	bioreactor	36
*Vampirovibrio chlorellavorus*	51.4	2,844	100%	3.0	parasite of *Chlorella*	37

a Genome completeness was estimated based on 83 single copy gene markers proposed by Soo *et al.* (37)

b Predicted genome size in parentheses.
