# Supplementary material for: Phylogenetic Diversity and Single-Cell Genome Analysis of “Melainabacteria”, a Non-Photosynthetic Cyanobacterial Group, in the Termite Gut
Source: Microbes Environ. 2018 Mar 29;33(1):50–7. doi: 10.1264/jsme2.ME17137 (PMC5877343; doi:10.1264/jsme2.ME17137)
Supplement: Supplementary file 1 [file 33_50_s1.pdf]

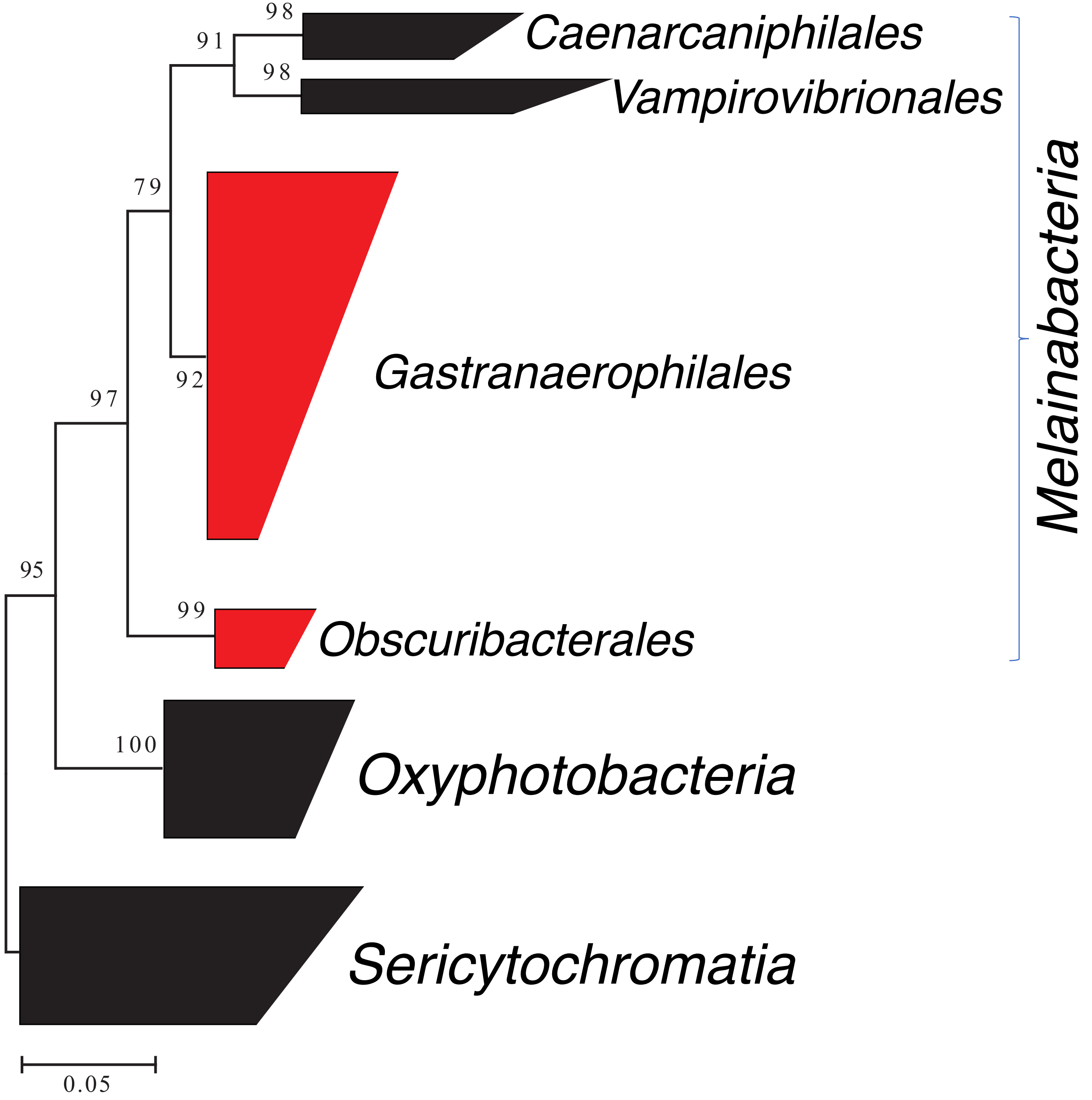

Fig. S1. Maximum likelihood tree based on near-full length 16S rRNA genes (>1,200 bp) from the phylum *Cyanobacteria*. The tree was constructed using program FastTree. A total of 1,096 nucleotide positions were used with the GTR+G substitution model and 100 bootstrap resamplings. Sequences from the termite and cockroach gut samples fell into two orders shown in red.

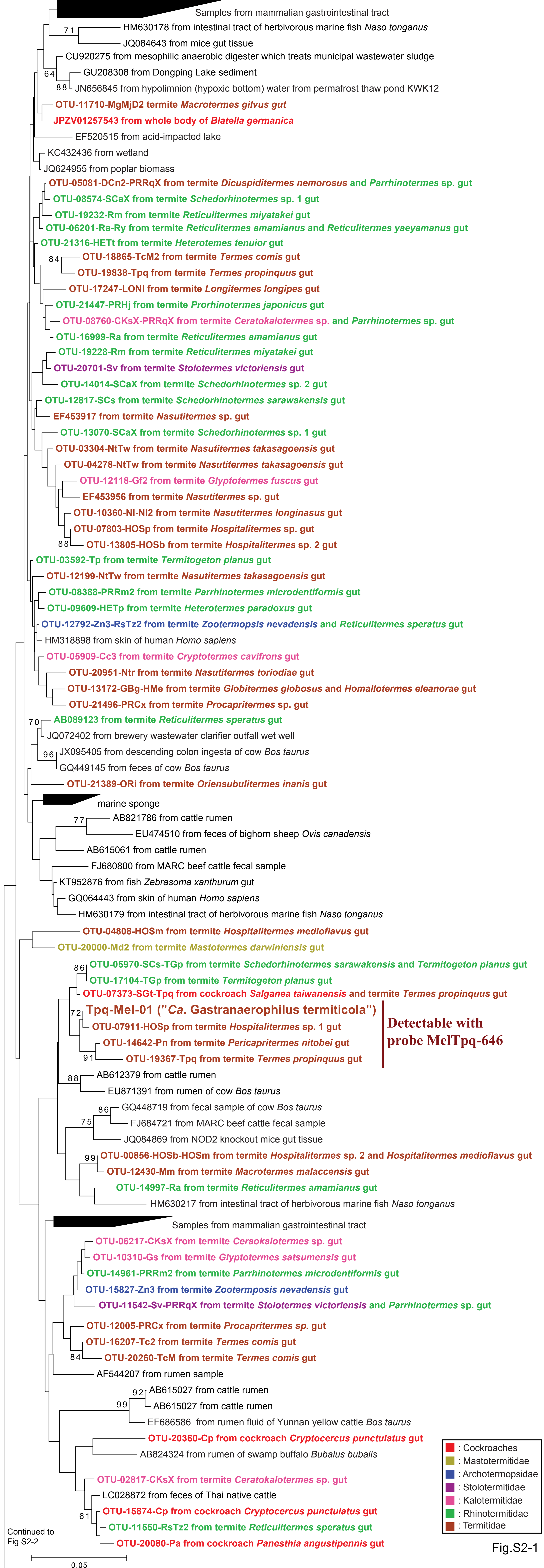

Samples from mammalian gastrointestinal tract
HM630178 from intestinal tract of herbivorous marine fish Naso tonganus
JQ084643 from mice gut tissue
CU920275 from mesophilic anaerobic digester which treats municipal wastewater sludge
GU208308 from Dongping Lake sediment
JN656845 from hypolimnion (hypoxic bottom) water from permafrost thaw pond KWK12
OTU-11710-MgMjD2 termite Macrotermes gilvus gut
JPZV01257543 from whole body of Blatella germanica
EF520515 from acid-impacted lake
KC432436 from wetland
JQ624955 from poplar biomass
OTU-05081-DCn2-PRRqX from termite Dicuspiditermes nemorosus and Parrhinotermes sp. gut
OTU-08574-SCaX from termite Schedorhinotermes sp. 1 gut
OTU-19232-Rm from termite Reticulitermes miyatakei gut
OTU-06201-Ra-Ry from termite Reticulitermes amamianus and Reticulitermes yaeyamanus gut
OTU-21316-HETt from termite Heterotemes tenuior gut
OTU-18865-TcM2 from termite Termes comis gut
OTU-19838-Tpq from termite Termes propinquus gut
OTU-17247-LONI from termite Longitermes longipes gut
OTU-21447-PRHj from termite Prorhinotermes japonicus gut
OTU-08760-CKsX-PRRqX from termite Ceratokalotermes sp. and Parrhinotermes sp. gut
OTU-16999-Ra from termite Reticulitermes amamianus gut
OTU-19228-Rm from termite Reticulitermes miyatakei gut
OTU-20701-Sv from termite Stolotermes victoriensis gut
OTU-14014-SCaX from termite Schedorhinotermes sp. 2 gut
OTU-12817-SCs from termite Schedorhinotermes sarawakensis gut
EF453917 from termite Nasutitermes sp. gut
OTU-13070-SCaX from termite Schedorhinotermes sp. 1 gut
OTU-03304-NtTw from termite Nasutitermes takasagoensis gut
OTU-04278-NtTw from termite Nasutitermes takasagoensis gut
OTU-12118-Gf2 from termite Glyptotermes fuscus gut
EF453956 from termite Nasutitermes sp. gut
OTU-10360-NI-NI2 from termite Nasutitermes longinasus gut
OTU-07803-HOSp from termite Hospitalitermes sp. gut
OTU-13805-HOSb from termite Hospitalitermes sp. 2 gut
OTU-03592-Tp from termite Termitogeton planus gut
OTU-12199-NtTw from termite Nasutitermes takasagoensis gut
OTU-08388-PRRm2 from termite Parrhinotermes microdentiformis gut
OTU-09609-HETp from termite Heterotermes paradoxus gut
OTU-12792-Zn3-RsTz2 from termite Zootermopsis nevadensis and Reticulitermes speratus gut
HM318898 from skin of human Homo sapiens
OTU-05909-Cc3 from termite Cryptotermes cavifrons gut
OTU-20951-Ntr from termite Nasutitermes toriodiae gut
OTU-13172-GBg-HMe from termite Globitermes globosus and Homallotermes eleanorae gut
OTU-21496-PRCx from termite Procapritermes sp. gut
AB089123 from termite Reticulitermes speratus gut
JQ072402 from brewery wastewater clarifier outfall wet well
JX095405 from descending colon ingesta of cow Bos taurus
GQ449145 from feces of cow Bos taurus
OTU-21389-ORi from termite Oriensubulitermes inanis gut
marine sponge
AB821786 from cattle rumen
EU474510 from feces of bighorn sheep Ovis canadensis
AB615061 from cattle rumen
FJ680800 from MARC beef cattle fecal sample
KT952876 from fish Zebrasoma xanthurum gut
GQ064443 from skin of human Homo sapiens
HM630179 from intestinal tract of herbivorous marine fish Naso tonganus
OTU-04808-HOSm from termite Hospitalitermes medioflavus gut
OTU-20000-Md2 from termite Mastotermes darwiniensis gut
OTU-05970-SCs-TGp from termite Schedorhinotermes sarawakensis and Termitogeton planus gut
OTU-17104-TGp from termite Termitogeton planus gut
OTU-07373-SGt-Tpq from cockroach Salganea taiwanensis and termite Termes propinquus gut
Tpq-Mel-01 ("Ca. Gastranaerophilus termiticola")
OTU-07911-HOSp from termite Hospitalitermes sp. 1 gut
OTU-14642-Pn from termite Pericapritermes nitobei gut
OTU-19367-Tpq from termite Termes propinquus gut
Detectable with probe MelTpq-646
AB612379 from cattle rumen
EU871391 from rumen of cow Bos taurus
GQ448719 from fecal sample of cow Bos taurus
FJ684721 from MARC beef cattle fecal sample
JQ084869 from NOD2 knockout mice gut tissue
OTU-00856-HOSb-HOSm from termite Hospitalitermes sp. 2 and Hospitalitermes medioflavus gut
OTU-12430-Mm from termite Macrotermes malaccensis gut
OTU-14997-Ra from termite Reticulitermes amamianus gut
HM630217 from intestinal tract of herbivorous marine fish Naso tonganus
Samples from mammalian gastrointestinal tract
OTU-06217-CKsX from termite Ceraokalotermes sp. gut
OTU-10310-Gs from termite Glyptotermes satsumensis gut
OTU-14961-PRRm2 from termite Parrhinotermes microdentiformis gut
OTU-15827-Zn3 from termite Zootermposis nevadensis gut
OTU-11542-Sv-PRRqX from termite Stolotermes victoriensis and Parrhinotermes sp. gut
OTU-12005-PRCx from termite Procapritermes sp. gut
OTU-16207-Tc2 from termite Termes comis gut
OTU-20260-TcM from termite Termes comis gut
AF544207 from rumen sample
AB615027 from cattle rumen
AB615027 from cattle rumen
EF686586 from rumen fluid of Yunnan yellow cattle Bos taurus
OTU-20360-Cp from cockroach Cryptocercus punctulatus gut
AB824324 from rumen of swamp buffalo Bubalus bubalis
OTU-02817-CKsX from termite Ceratokalotermes sp. gut
LC028872 from feces of Thai native cattle
OTU-15874-Cp from cockroach Cryptocercus punctulatus gut
OTU-11550-RsTz2 from termite Reticulitermes speratus gut
OTU-20080-Pa from cockroach Panesthia angustipennis gut
: Cockroaches
: Mastotermitidae
: Archotermopsidae
: Stolotermitidae
: Kalotermitidae
: Rhinotermitidae
: Termitidae
Continued to Fig.S2-2
0.05

Fig.S2-1

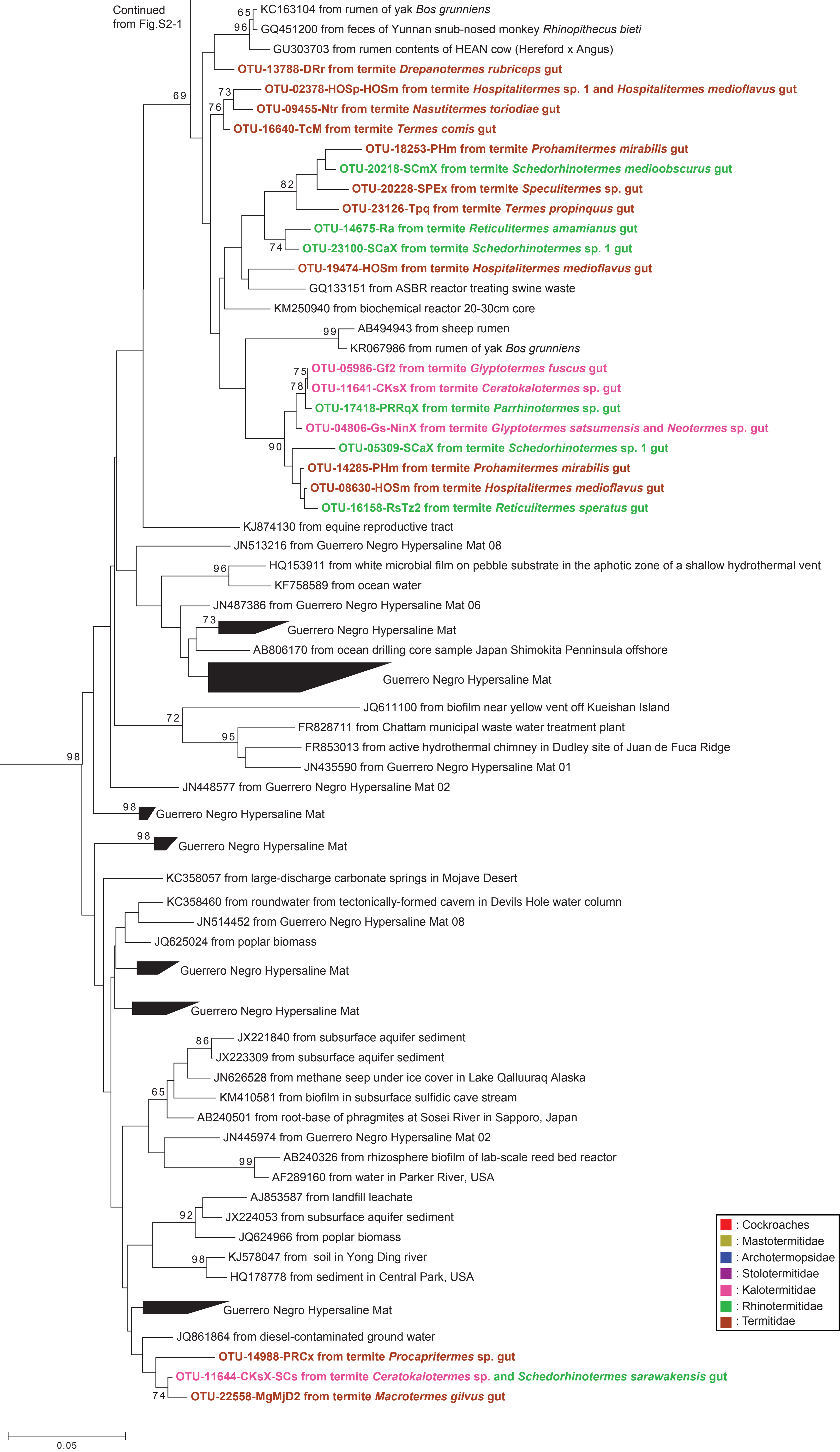

Fig. S2-2. Phylogenetic positions of 16S rRNA gene sequences affiliated with "*Gastranaerophilales*", obtained from termite and cockroach samples. Taxa of the host insects are shown in indicated colors. A neighbor-joining tree was constructed using a total of 306 nucleotide positions with the Jukes-Cantor substitution model and 100 bootstrap resamplings. Only bootstrap confidence values ≥60% are shown. Sequences of "*Obscuribacterales*" were used as outgroup (see Fig. S3).

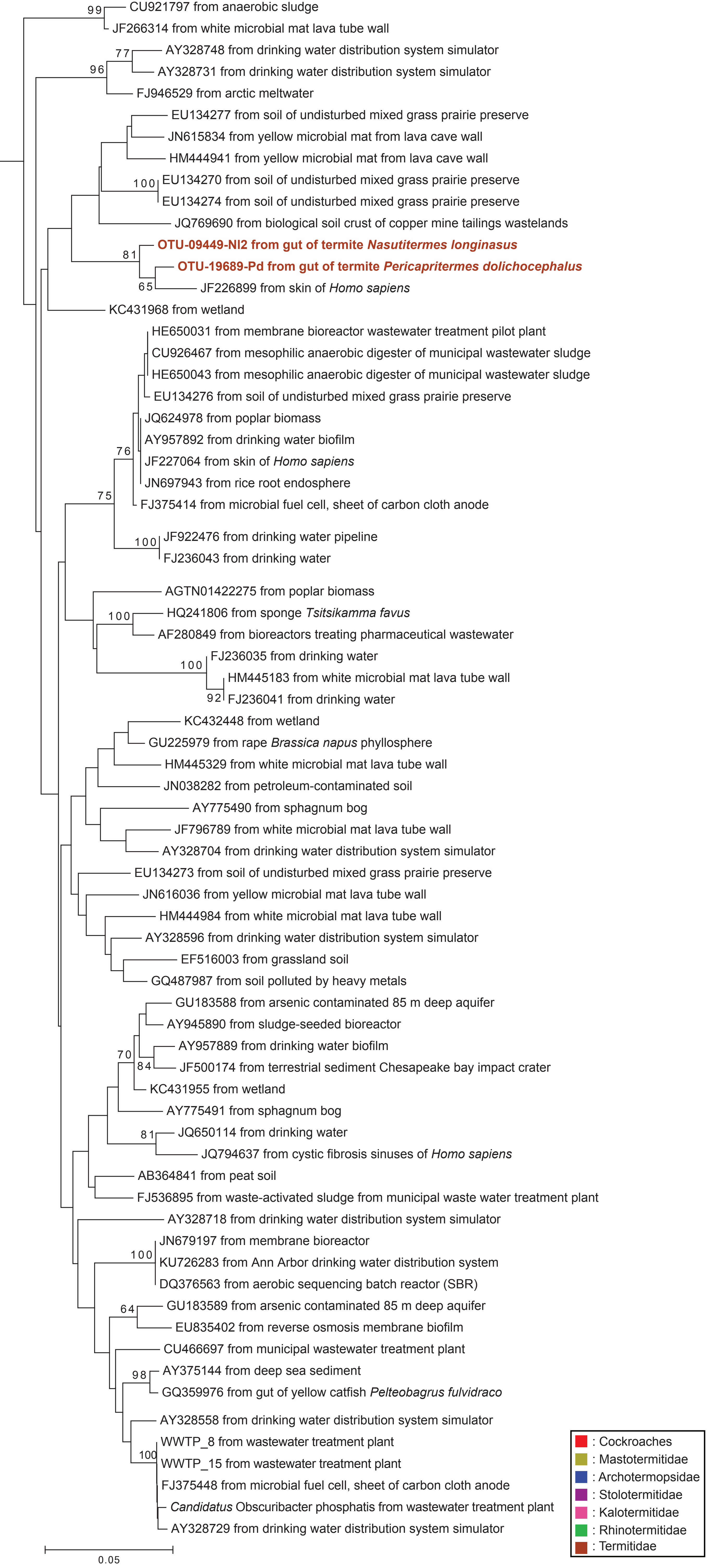

Fig. S3. Phylogenetic positions of 16S rRNA gene sequences affiliated with "*Obscuribacterales*", obtained from termite and cockroach samples. Sequences of "*Gastranaerophilales*" were used as outgroups (see Fig. S2). See the legend to Fig. S2 for explanation.

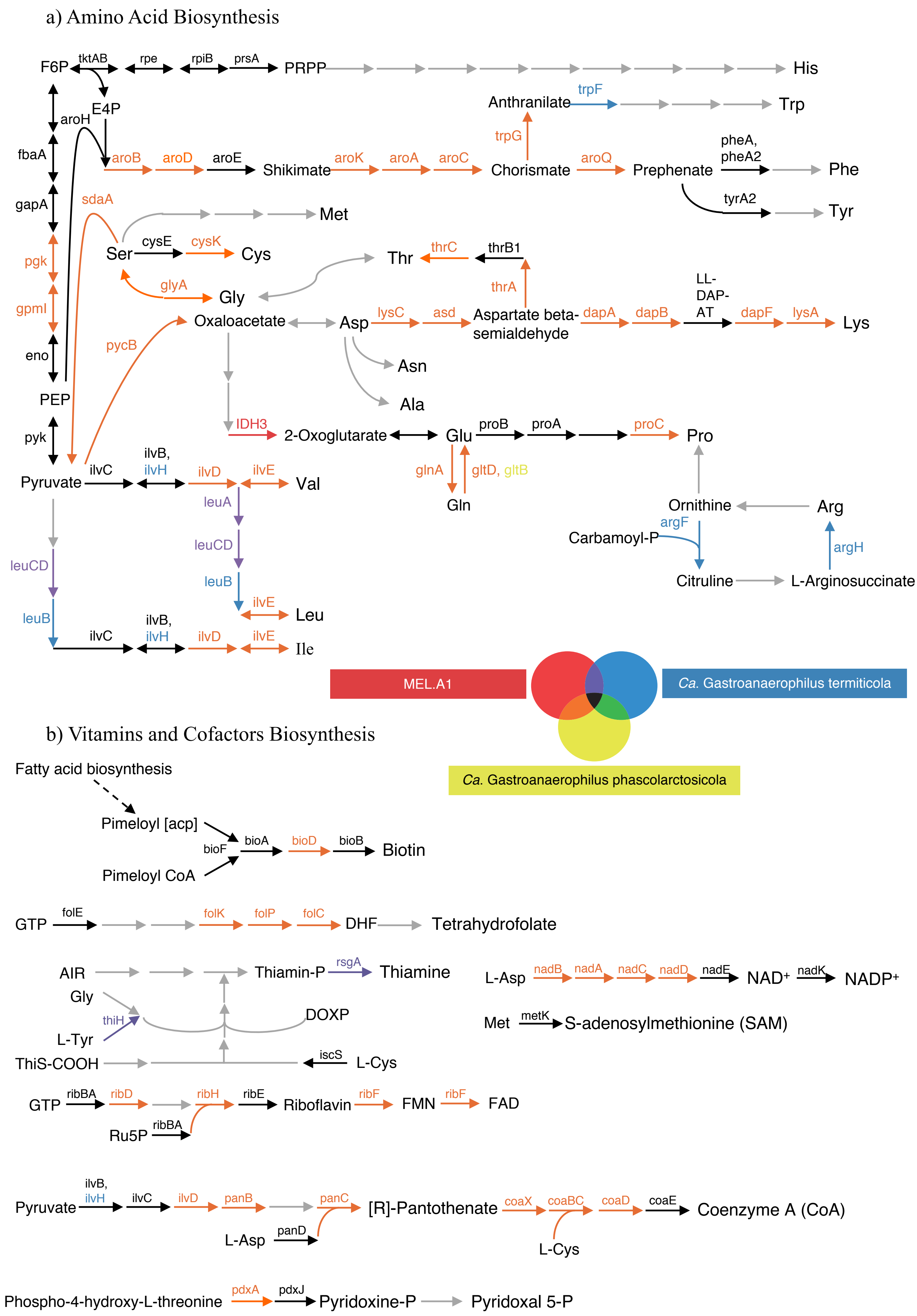

Fig. S4. Pathways for amino acid and cofactor biosynthesis found from the Tpq-Mel-01 genome, shown in comparison with "*Ca*. Gastranaerophilus phascolarctosicola" and MEL.A1. Components found in one or more genomes are highlighted with colors indicated in the top right corner. Missing pathways or components are shown in gray.

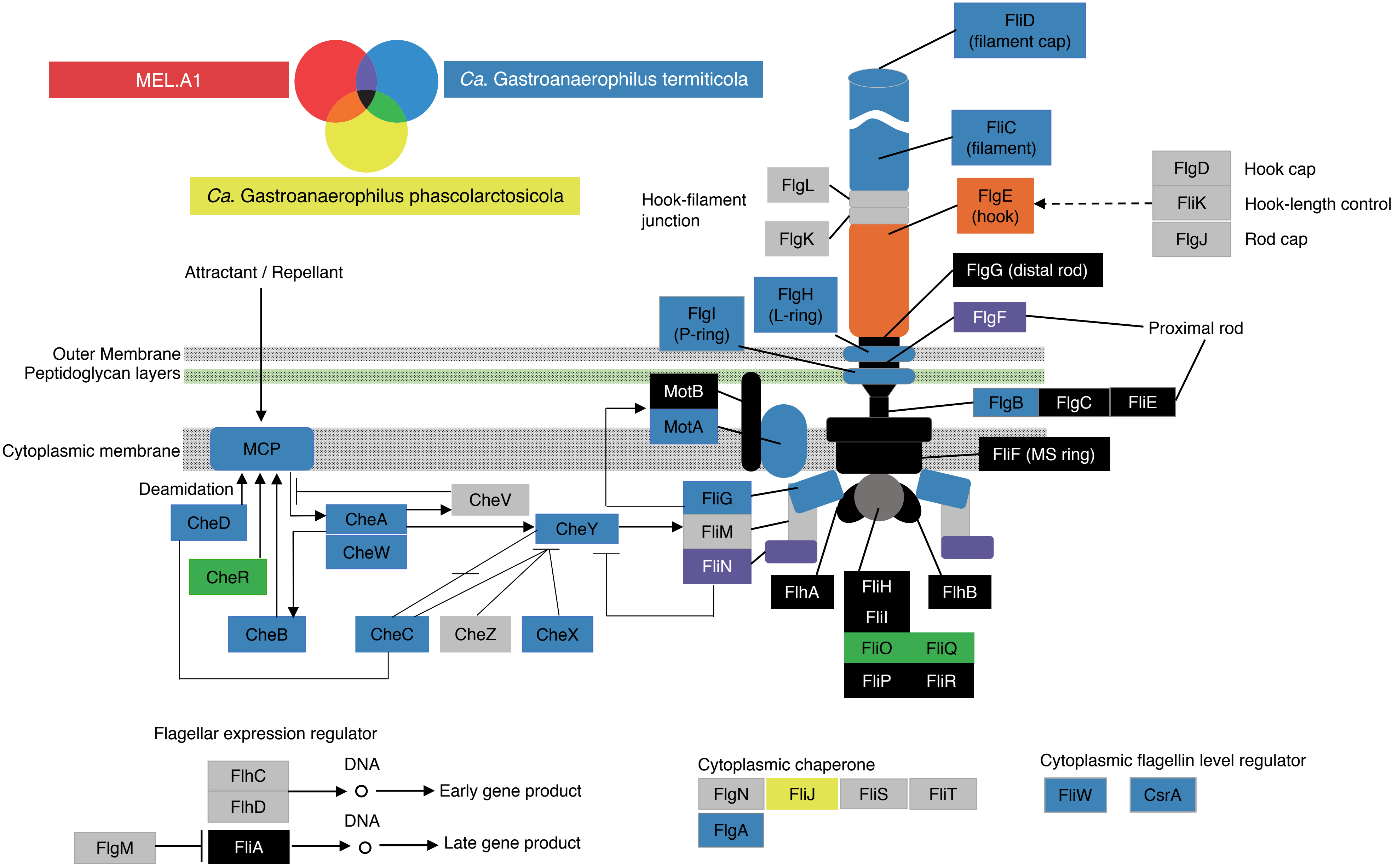

Fig. S5. Genes coding for flagellar components found from the Tpq-Mel-01 genome, shown in comparison with "*Ca*. Gastranaerophilus phascolarctosicola" and MEL.A1. See the legend to Fig. S4 for explanation.

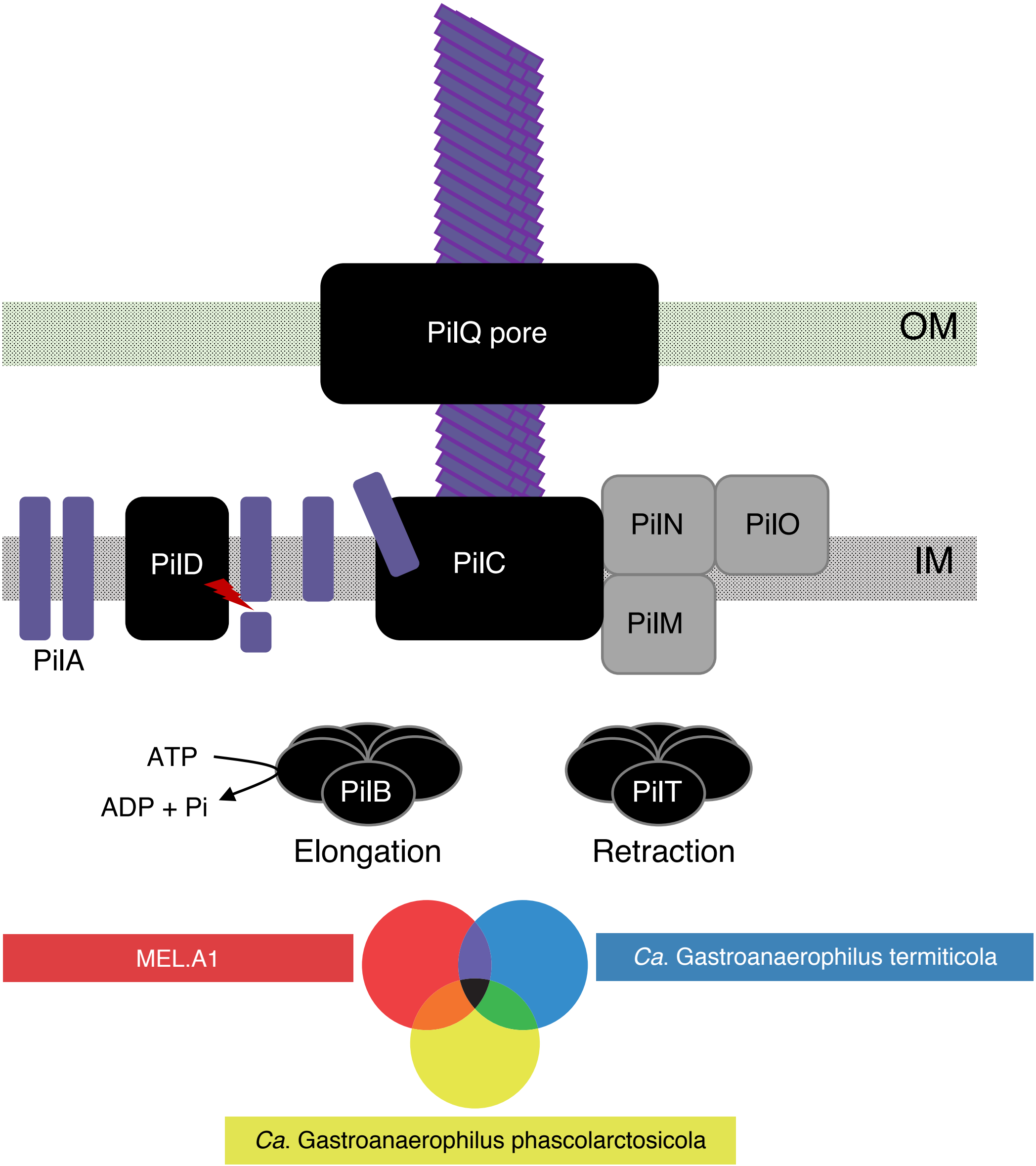

Fig. S6. Genes coding for pilus components found from the Tpq-Mel-01 genome, shown in comparison with "*Ca*. Gastranaerophilus phascolarctosicola" and MEL.A1. See the legend to Fig. S4 for explanation.

Table S1. List of termites and cockroaches used in this study.

(Sub) family of host insects	Species of host insects	OTU code	Origin	Feeding-type	Total number of reads	Total number of melainabacterial reads	Number of melainabacterial OTUs
Ectobiidae	*Blatella germanica*	Bg	Japan	omnivorous	9,717	0	0
Blattellidae	*Symploce gigas*	SYg	Japan	omnivorous	726,304	0	0
Blattidae	*Periplaneta americana*	PPam	Japan	omnivorous	17,668	0	0
Blattidae	*Periplaneta japonica*	PPja	Japan	omnivorous	38,006	0	0
Blaberidae	*Opisthoplatia orientalis*	OPo	Japan	omnivorous	15,254	0	0
Blaberidae	*Salganea taiwanensis*	SGt	Japan	wood feeder	14,639	14	1
Blaberidae	*Panesthia angustipennis*	Pa	Japan	wood feeder	9,340	11	1
Cryptocercidae	*Cryptocercus punctulatus*	Cp	USA	wood feeder	13,295	30	2
Mastotermitidae	*Mastotermes darwiniensis*	Md2	Australia	wood feeder	23,141	11	1
Archotermopsidae	*Archotermopsis wroughtoni*	APK	Thailand	wood feeder	28,782	0	0
Archotermopsidae	*Hodotermopsis sjoestedti*	Hs3	Japan	wood feeder	23,253	0	0
Archotermopsidae	*Hodotermopsis* sp.	HsK	Japan	wood feeder	24,306	0	0
Archotermopsidae	*Zootermopsis nevadensis*	Zn3	Japan	wood feeder	31,667	42	2
Stolotermitidae	*Stolotermes victoriensis*	Sv	Australia	wood feeder	27,786	26	2
Kalotermitidae	*Ceratokalotermes* sp.	CKsX	Australia	wood feeder	27,227	312	5
Kalotermitidae	*Glyptotermes satsumensis*	Gs	Japan	wood feeder	24,501	62	2
Kalotermitidae	*Glyptotermes fuscus*	Gf2	Japan	wood feeder	62,143	107	2
Kalotermitidae	*Neotermes* sp.	NinX	Australia	wood feeder	44,993	72	1
Kalotermitidae	*Neotermes koshunensis*	Nk	Japan	wood feeder	18,466	0	0
Kalotermitidae	*Neotermes koshunensis*	Nk2	Japan	wood feeder	23,944	0	0
Kalotermitidae	*Neotermes koshunensis*	Nk3	Japan	wood feeder	25,988	0	0
Kalotermitidae	*Cryptotermes cavifrons*	Cc3	USA	wood feeder	36,621	76	1
Rhinotermitidae	*Parrhinotermes microdentiformis*	PRRm	Malaysia	wood feeder	37,584	0	0
Rhinotermitidae	*Parrhinotermes microdentiformis*	PRRm2	Malaysia	wood feeder	44,523	75	2

Rhinotermitidae	*Parrhinotermes* sp.	PRRqX	Australia	wood feeder	42,692	121	4
Rhinotermitidae	*Schedorhinotermes medioobscurus*	SCm	Thailand	wood feeder	31,486	0	0
Rhinotermitidae	*Schedorhinotermes* sp. 2	SCmX	Malaysia	wood feeder	36,273	11	1
Rhinotermitidae	*Schedorhinotermes sarawakensis*	SCs	Malaysia	wood feeder	23,946	98	3
Rhinotermitidae	*Schedorhinotermes* sp. 1	SCaX	Australia	wood feeder	25,552	195	5
Rhinotermitidae	*Prorhinotermes japonicus*	PRHj	Taiwan	wood feeder	33,261	8	1
Rhinotermitidae	*Coptotermes formosanus*	Cf3	Japan	wood feeder	34,332	0	0
Rhinotermitidae	*Coptotermes curvignathus*	COPc	Malaysia	wood feeder	28,349	0	0
Rhinotermitidae	*Heterotermes paradoxus*	HETp	Australia	wood feeder	48,162	44	1
Rhinotermitidae	*Heterotermes tenuior*	HETt	Malaysia	wood feeder	44,055	8	1
Rhinotermitidae	*Reticulitermes amamianus*	Ra	Japan	wood feeder	36,123	124	4
Rhinotermitidae	*Reticulitermes miyatakei*	Rm	Japan	wood feeder	36,529	26	2
Rhinotermitidae	*Reticulitermes* sp. RPK	RPK2	Thailand	wood feeder	45,685	0	0
Rhinotermitidae	*Reticulitermes speratus*	RsTz2	Japan	wood feeder	42,643	64	3
Rhinotermitidae	*Reticulitermes yaeyamanus*	Ry	Taiwan	wood feeder	26,746	14	1
Rhinotermitidae	*Termitogeton planus*	TGp	Malaysia	wood feeder	55,608	167	3
Termitidae							
Macrotermitinae	*Macrotermes gilvus*	MgMjD2	Thailand	fungus grower	12,902	39	2
Macrotermitinae	*Macrotermes malaccensis*	Mm	Malaysia	fungus grower	31,586	31	1
Macrotermitinae	*Odontotermes formosanus*	Of	Japan	fungus grower	26,153	0	0
Apicotermitinae	*Speculitermes* sp.	SPEx	Thailand	soil-grass feeder	25,021	11	1
Amitermitinae	*Amitermes longignathus*	Alo	Thailand	soil feeder	29,953	0	0
Amitermitinae	*Amitermes laurensis*	Ala	Australia	grass feeder	15,484	0	0
Amitermitinae	*Drepanotermes rubriceps*	DRr	Australia	grass feeder	27,366	26	1
Termitinae	*Microcerotermes dubius*	MCd	Malaysia	wood feeder	17,537	0	0
Termitinae	*Microcerotermes* sp. M1NP1	M1PT4b	Thailand	wood feeder	24,921	0	0
Termitinae	*Microcerotermes crassus*	McPP3	Thailand	wood feeder	41,609	0	0
Termitinae	*Globitermes globosus*	GBg	Malaysia	wood feeder	27,963	20	1
Termitinae	*Prohamitermes mirabilis*	PHm	Malaysia	interface feeder	24,752	40	2

Termitinae	*Prohamitermes mirabilis*	PHm2	Malaysia	interface feeder	3,258	0	0
Termitinae	*Termes comis*	Tc2	Thailand	interface feeder	16,746	20	1
Termitinae	*Termes comis*	TcM	Malaysia	interface feeder	19,226	30	2
Termitinae	*Termes comis*	TcM2	Malaysia	interface feeder	11,295	14	1
Termitinae	*Termes propinquus*	Tpq	Thailand	interface feeder	17,190	74	4
Termitinae	*Macrognathotermes errator*	MGe	Australia	interface feeder	21,704	0	0
Termitinae	*Homallotermes eleanorae*	HMe	Malaysia	interface feeder	19,816	8	1
Termitinae	*Pericapritermes nitobei*	Pn	Japan	soil feeder	28,119	24	1
Termitinae	*Pericapritermes dolichocephalus*	Pd	Malaysia	soil feeder	11,069	12	1
Termitinae	*Procapritermes* sp.	PRCx	Malaysia	soil feeder	24,929	64	3
Termitinae	*Procapritermes setiger*	PRCs	Malaysia	soil feeder	15,793	0	0
Termitinae	*Dicuspiditermes nemorosus*	DCn	Malaysia	soil feeder	16,236	0	0
Termitinae	*Dicuspiditermes nemorosus*	DCn2	Malaysia	soil feeder	13,104	27	1
Nasutitermitinae	*Nasutitermes dimorphus*	Nd	Thailand	wood feeder	37,635	0	0
Nasutitermitinae	*Nasutitermes longinasus*	Nl	Malaysia	wood feeder	43,659	24	1
Nasutitermitinae	*Nasutitermes longinasus*	Nl2	Malaysia	wood feeder	92,889	61	2
Nasutitermitinae	*Nasutitermes takasagoensis*	NtTw	Taiwan	wood feeder	81,643	276	3
Nasutitermitinae	*Nasutitermes takasagoensis*	Nt2	Japan	wood feeder	26,265	0	0
Nasutitermitinae	*Nasutitermes toriodiae*	Ntr	Australia	grass feeder	34,551	54	2
Nasutitermitinae	*Hospitalitermes medioflavus*	HOSm	Malaysia	lichen feeder	35,027	665	5
Nasutitermitinae	*Hospitalitermes* sp. 1	HOSp	Thailand	lichen feeder	23,144	150	3
Nasutitermitinae	*Hospitalitermes* sp. 2	HOSb	Malaysia	lichen feeder	23,834	147	2
Nasutitermitinae	*Oriensubulitermes inanis*	ORi	Malaysia	soil feeder	26,105	8	1
Nasutitermitinae	*Leucopitermes leucopus*	LEUl	Malaysia	wood feeder	18,486	0	0
Nasutitermitinae	*Longipeditermes longipes*	LONl	Malaysia	grass-leaf feeder	50,378	17	1

Table S2. PCR primers and FISH probes used in this study.

Primer/Probe ID	Sequence	Reference
COII-FW-A-tLeu	5′-CAGATAAGTGCATTGGATTT-3′	4
COII-RV-B-tLys	5′-GTTTAAGAGACCAGTACTTG-3′	4
27F-mix	5′-AGRGTTTGATYMTGGCTCAG-3′	2
1390R	5′-ACGGGCGGTGTGTACAA-3′	2
341F	5′-AATGATACGGCGACCACCGAGATCTACAC-3′	3
806R	5′-AGTCAGTCAGCCGGACTACHVGGGTWTCTAAT-3′	3
341F-read1	5′-TATGGTAATTGGCCTACGGGAGGCAGCAG-3′	3
806R-read2	5′-AGTCAGTCAGCCGGACTACHVGGGTWTCTAAT-3′	3
MelTpq-646 (Texas Red)	5′-TACCACATTCTAGTTTAT-3′	present study
EUB338 (6FAM Green)	5′-GCTGCCTCCCGTAGGAGT-3′	1

Table S3. List of bacteria used in the phylogenomic tree shown in Fig. 2 and their genomic distance (nucleotide sequence dissimilarity of aligned genome regions) to phylotype Tpq-Mel-01 (“*Ca*. Gastranaerophilales termiticola”) based on calculation using Genome-to-Genome Distance Calculator.

Class	Strain	Genome distance	Accession number
Oxyphotobacteria	*Gloeobacter violaceus* PCC 7421	0.18	GCF_000011385.1
	Calothrix sp. PCC 7507	0.20	GCA_000316575.1
	Leptolyngbya sp. PCC 7376	0.19	GCA_000316605.1
	Synechococcus elongatus PCC 7942	0.23	GCA_000012525.1
	Prochlorococcus sp. MIT 0801	0.23	GCA_000757865.1
Melainabacteria	*Vampirovibrio chlorellavorus*	0.17	GCA_001858525
	ACD20	0.11	GCA_000299275
	Candidatus Melainabacteria bacterium MEL.A1	0.11	GCA_001765415
	Candidatus Melainabacteria bacterium MEL.B2	0.10	*MEL.B2
	Candidatus Melainabacteria bacterium MEL.C1	0.11	*MEL.C1
	Candidatus Gastranaerophilales bacterium MH_37	0.12	GCA_001899425
	Candidatus Gastranaerophilus phascolarctosicola	0.07	GCA_001899335
	Candidatus Gastranaerophilales bacterium Zag_1	0.13	GCA_001899365
	Candidatus Gastranaerophilales bacterium Zag_111	0.14	GCA_001899395
	Candidatus Gastranaerophilales bacterium HUM_1	0.07	GCA_002103105
	Candidatus Gastranaerophilales bacterium HUM_2	0.14	GCA_002103055
	Candidatus Gastranaerophilales bacterium HUM_3	0.11	GCA_002102975
	Candidatus Gastranaerophilales bacterium HUM_4	0.12	GCA_002102985
	Candidatus Gastranaerophilales bacterium HUM_5	0.12	GCA_002103085
	Candidatus Gastranaerophilales bacterium HUM_6	0.10	GCA_002102805
	Candidatus Gastranaerophilales bacterium HUM_7	0.08	GCA_002102735
	Candidatus Gastranaerophilales bacterium HUM_8	0.08	GCA_002102725
	Candidatus Gastranaerophilales bacterium HUM_9	0.15	GCA_002103075
	Candidatus Gastranaerophilales bacterium HUM_10	0.12	GCA_002102745
	Candidatus Gastranaerophilales bacterium HUM_11	0.11	GCA_002102995
	Candidatus Gastranaerophilales bacterium HUM_12	0.12	GCA_002102825
	Candidatus Gastranaerophilales bacterium HUM_13	0.14	GCA_002102875
	Candidatus Gastranaerophilales bacterium HUM_15	0.11	GCA_002102815
	Candidatus Gastranaerophilales bacterium HUM_16	0.09	GCA_002102885
	Candidatus Gastranaerophilales bacterium HUM_17	0.08	GCA_002102905
	Candidatus Gastranaerophilales bacterium HUM_18	0.11	GCA_002102715
	Candidatus Gastranaerophilales bacterium HUM_21	0.09	GCA_002103135
	Candidatus Gastranaerophilales bacterium HUM_22	0.07	GCA_002103035
	Candidatus Gastranaerophilales bacterium HUM_23	0.12	GCA_002102895
	CAG_196	0.11	GCA_000433235
	CAG_306	0.11	GCA_000431555

	CAG_439	0.18	GCA_000438175
	CAG_484	0.12	GCA_000431315
	CAG_715	0.11	GCA_000431115
	CAG_815	0.08	GCA_000437775
	Candidatus Obscuribacter phosphatis	0.19	GCA_001899315
	WWTP_8	0.17	**3300003765
	WWTP_15	0.17	**3300003764
	Candidatus Caenarcanum bioreactoricola	0.23	GCA_001899385
	SSGW_16	0.22	**3300000574
Sericytochromatia	CBMW_12	0.17	GCA_002083825
	RAAC_196	0.09	GCA_002083785
	LSPB_72	1.00	GCA_002083815

*) ggkBase accession names not deposited in Genbank.
**) IMG accession numbers not deposited in Genbank.

References for supplementary tables

1. Amann, R.I., B.J. Binder, R.J. Olson, S.W. Chisholm, R. Devereux, and D.A. Stahl. 1990. Combination of 16S rRNA-targeted oligonucleotide probes with flow cytometry for analyzing mixed microbial populations. Appl. Environ. Microbiol. 56:1919–1925.
2. Hongoh, Y., T. Sato, M.F. Dolan, S. Noda, S. Ui, T. Kudo, and M. Ohkuma. 2007. The motility symbiont of the termite gut flagellate *Caduceia versatilis* is a member of the "*Synergistes*" group. Appl. Environ. Microbiol. 73:6270–6276.
3. Kozich, J.J., S.L. Westcott, N.T. Baxter, S.K. Highlander, and D. Patrick. 2013. Development of a dual-index sequencing strategy and curation pipeline for analyzing amplicon sequence data on the MiSeq Illumina sequencing platform. Appl. Environ. Microbiol. 79:5112–5120.
4. Miura T., K. Maekawa, O. Kitade, T. Abe, and T. Matsumoto. 1998. Phylogenetic relationships among subfamilies in higher termites (Isoptera: Termitidae) based on mitochondrial COII gene sequences. Ann. Entomol. Soc. Am. 91:515–521.
